# The Long-Term Effects of Using Phosphate-Solubilizing Bacteria and Photosynthetic Bacteria as Biofertilizers on Peanut Yield and Soil Bacteria Community

**DOI:** 10.3389/fmicb.2021.693535

**Published:** 2021-07-16

**Authors:** Yiming Wang, Shuang Peng, Qingqing Hua, Chongwen Qiu, Pan Wu, Xiaoli Liu, Xiangui Lin

**Affiliations:** ^1^State Key Laboratory of Soil and Sustainable Agriculture, Institute of Soil Science, Chinese Academy of Sciences, Nanjing, China; ^2^Jiangsu Collaborative Innovation Center for Solid Organic Waste Resource Utilization, Nanjing, China; ^3^College of Environment and Ecology, Jiangsu Open University, Nanjing, China; ^4^National Engineering and Technology Research Center for Red Soil Improvement, Experimental Station of Red Soil, Chinese Academy of Sciences, Yingtan, China

**Keywords:** phosphate solubilizing bacteria, purple non-sulfur bacteria, peanut yield, bacteria community, soil microbial P-transformation

## Abstract

Microbial inoculation is a promising strategy to improve crop yields and reduce the use of chemical fertilizers, thereby creating environment-friendly agriculture. In this study, the long-term (5 years) effects of a phosphate-solubilizing bacterium *Burkholderia cepacia* ISOP5, a purple non-sulfur bacterium *Rhodopseudomonas palustris* ISP-1, and a mixed inoculation of these two bacteria (MB) on peanut yield, soil microbial community structure, and microbial metabolic functions were evaluated in a field experiment. After 5 years of inoculation, total peanut yield with *B. cepacia* ISOP5, *R. palustris* ISP-1, and MB treatments increased by 8.1%, 12.5%, and 19.5%, respectively. The treatments also significantly promoted the absorption of N and increased the protein content in peanut seeds. Nutrient content also increased to some extent in the bacteria-inoculum-treated soil. However, bacterial community diversity and richness were not significantly affected by bacterial inoculums, and only minor changes occurred in the bacterial community composition. Functional prediction revealed that bacterial inoculums reduced the relative abundance of those genes associated with P uptake and transport as well as increased the abundance of genes associated with inorganic P solubilization and organic P mineralization. Bacterial inoculums also increased the total relative abundance of genes associated with N metabolism. In addition to developing sustainable and eco-friendly agricultural practice, crop inoculation with *B. cepacia* ISOP5 and *R. palustris* ISP-1 would improve soil fertility, enhance microbial metabolic activity, and increase crop yield.

## Introduction

Plants are closely associated with a wide variety of bacterial taxa, which play crucial roles in plant growth, disease prevention, and stress tolerance. Among these beneficial bacteria, plant growth-promoting bacteria (PGPB) are the most extensively studied (Lee et al., 2008). These bacteria have been isolated from the rhizosphere, endosphere, and phyllosphere of a wide variety of plant species (Rilling et al., 2019). PGPB can improve soil fertility and enhance plant nutrient uptake either by providing essential nutrients such as phosphorus (P) and nitrogen (N) and essential minerals, or by synthesizing phytohormones (Valetti et al., 2018). Therefore, the combined use of PGPB in the form of biofertilizers has been regarded as a promising method to reduce chemical fertilizer usage while sustaining soil fertility and increasing crop yield (Wong et al., 2014). This, in turn, can make agriculture more sustainable and productive.

Phosphate-solubilizing bacteria (PSB) and purple phototrophic bacteria (PPB) are two kinds of PGPB. Phosphorus is an essential nutrient for plant growth and agricultural production, but the majority of P is unavailable to plants (Wu et al., 2019). PSB such as *Bacillus*, *Burkholderia*, *Azospirillum*, *Erwinia*, *Pseudomonas*, *Rhizobium*, and *Serratia* can convert insoluble P into soluble forms that are absorbable by plants and thus enhance plant growth (Ramakrishna et al., 2019; Zhang et al., 2019). Therefore, PSB play a crucial role in the transformation and biogeochemical cycling of insoluble and soluble P in agroecosystems (Li F. et al., 2017; Yu et al., 2019). Inoculation with PSB as biofertilizers has been reported to increase P uptake (Rezakhani et al., 2019), improve leaf N and P contents (Wu et al., 2019), promote plant growth (Panhwar et al., 2014; Parastesh et al., 2019), and aid in increasing crop yield (Jaybhay et al., 2017). Thus, microbial intervention of high-efficiency PSB seems to be an effective way to solve the problem of P availability in soil and avert the negative effects of chemical P fertilizers.

The ability of PSB to mineralize organic P and solubilize inorganic and insoluble phosphates varies among different types of bacteria as well as with different chemical traits of the medium (Parastesh et al., 2019). In addition, when PSB is used as a biofertilizer in the field, crops respond differently due to the influence of soil temperature, moisture, pH, salinity, sources of insoluble P, sources of energy, inoculation methods, and the microorganism strains used (Sharma et al., 2013). Numerous bacterial strains have promising results at the laboratory scale and show promise as biofertilizers or inoculants (Rilling et al., 2019). However, consistent responses under agronomic conditions are lacking, owing to the different climatic and management conditions in the laboratory versus those in the field. Therefore, there is an urgent need to investigate the effects of selected high-efficiency PSB on plant growth and crop yield in field applications and their long-term effects.

PPB are a group of bacteria that can interact with plants to promote plant growth (Harada et al., 2005). They are widely distributed in natural environments such as oceans, lakes, sediments, and soils (Feng et al., 2011). PPB are a group of dinitrogen (N_2_)-fixing bacteria (Ludden and Roberts, 2002), which includes purple non-sulfur bacteria (PNSB) and purple sulfur bacteria (Feng et al., 2009). PNSB are a group of polyphosphate-accumulating organisms (Liang et al., 2010) that can excrete phytohormones to enhance plant growth (Kantha et al., 2015). Most species of PNSB, such as *Rhodopseudomonas* spp., are able to grow aerobically in the dark or anaerobically in the light, with many carbon sources and electron donors (Lee et al., 2008). Applications of PNSB in agriculture, particularly *Rhodopseudomonas palustris*, have been found to increase rice yields by 29% (Harada et al., 2005) and *Stevia rebaudiana* yields by 69.2% (Wu et al., 2013). Moreover, PNSB can produce bioactive compounds such as herbicides, antiviral substances, and antimicrobial compounds that inhibit plant pathogenic microbes (Kantha et al., 2015). As one of the oldest photosynthetic bacteria on earth, *R. palustris* has been reported to produce many chemicals, including riboflavin, iron carriers, 5-aminolevulinic acid, exopolysaccharides (EPS), and bacterial acyl-high serine lactone that trigger induced disease resistance in plants (Zhai et al., 2021). In view of these aspects, PNSB could also be used as a beneficial biofertilizer in agriculture.

Despite the multiple functional properties of PSB and PPB that enhance plant growth, they are yet to fulfill their potential as commercial bioinoculants. Although studies have shown that inoculation of PSB and PNSB can increase crop yields, it is unclear whether they can maintain a stable increase in yield during long-term application. In a previous study (Wu et al., 2013), we isolated a PNSB strain which can secrete EPS and promote plant growth and a PSB strain with a high capacity to dissolve insoluble phosphorus. We conducted a 5-year (2012–2016) field trial to observe the long-term impact of the PSB strain on peanut yield when used alone or in combination with the PNSB strain. The main objectives of this study were to investigate: (1) the yield-increasing effect of these strains in long-term fertilization when applied separately or in combination; (2) the influence of these inoculants on soil chemical properties after repeated application over 5 years; and (3) the influence of these inoculants on the bacterial community as well as the functional characteristics of bacterial communities that relate to P and N cycling in soil. The results of this study can help elucidate the effect of using bacterial inoculants as biofertilizers on yield increase, soil fertility, and the bacterial community in long-term agricultural production.

## Materials and Methods

### Preparation of Bacterial Inoculum Suspension

The PSB and PNSB under investigation were identified as *Burkholderia cepacia* strain ISOP5 (CCTCC M2020482) and *Rhodopseudomonas palustris* strain ISP-1 (Wu et al., 2013), respectively. The PSB were isolated from soil samples collected from peanut fields at the National Agro-Ecosystem Observation and Research Station in Yingtan (28°15′20″ N, 116°55′30″ E), Jiangxi Province, China. The strain *B. cepacia* ISOP5 was selected and purified for our field experiment on the basis of the appearance of a large clear halo on a plate of Tricalcium Phosphate medium [1 L distilled water with 10 g glucose, 0.5 g (NH_4_)_2_SO_4_, 0.3 g KCl, 0.1 g MgSO_4_⋅7H_2_O, 0.3 g NaCl, 0.03 g FeSO_4_⋅7H_2_O, 0.03 g MnSO_4_⋅4H_2_O, 10 g Ca_3_(PO_4_)_2_, and 20 g agar; pH 7.0–7.5] and Lecithin medium [constituents are the same as those of Tricalcium Phosphate medium, except for replacing the Ca_3_(PO_4_)_2_ with 5 g CaCO_3_ and 0.2 g lecithin]. The phosphorus-dissolving halo of *B. cepacia* ISOP5 is shown in Supplementary Figure 1. *R. palustris* ISP-1 was isolated from Xuanwu Lake, Nan Jing, Jiangsu Province, China. In our previous study, we found that this strain has the ability to promote plant growth; details were described in Wu et al. (2013).

To prepare the bacterial inoculums, we cultured *B. cepacia* ISOP5 in LB medium and incubated it at 28 ± 2°C for about 5 days (the stable growth phase) on a shaker with a rotation speed of 160 rpm. Meanwhile, *R. palustris* ISP-1 was cultured in 20 L chemical culture medium (Li et al., 2011) for about 20 days (for more EPS) at 30°C under a 60 W incandescent lamp placed at a distance of 25 mm. When the density of bacteria suspension reached about 10^10^ colony forming units (CFU) mL^–1^, the cells were harvested by centrifugation at 4000 × *g* for 20 min. Then the cells were washed twice with sterile deionized water and resuspended in sterile distilled water containing 0.8% (w/v) NaCl to achieve an optical density of 1.0 at 600 nm (OD600) corresponding to a bacterial density of about 10^9^ CFU mL^–1^. The pellets of *B. cepacia* ISOP5 and *R. palustris* ISP-1 were diluted 10 times with water to obtain a final concentration of 10^8^ CFU mL^–1^, and then the suspensions of *B. cepacia* ISOP5 and *R. palustris* ISP-1 were used for the field experiment.

### Site Description and Experimental Design

The long-term fertilization experiment was established in 2012 at the National Agro-Ecosystem Observation and Research Station in Yingtan (28°15′20″ N, 116°55′30″ E), Jiangxi Province, China. The soil, which was derived from Quaternary red clay, contained 36.3% clay, 42.5% silt, 21.2% sand, 41.8 g/kg total iron, and 76.6 g/kg total aluminum (Jiang et al., 2018). Monthly rainfall (Supplementary Figure 2) and air temperature (Supplementary Figure 3) were collected from the local weather station.

Five treatments were set up for the study: (1) conventional chemical fertilization containing 172.5 kg N ha^–1^, 90 kg P_2_O_5_ ha^–1^, and 180 kg K_2_O ha^–1^; urea (N 46%), fused calcium-magnesium phosphate (P_2_O_5_ 12%), and potassium chloride (K_2_O 60%) were applied as NPK fertilizers. (2) basic fertilizer (CK) containing 85.4 N ha^–1^, 70 kg P_2_O_5_ ha^–1^, and 115 kg K_2_O ha^–1^ plus 1500 kg/ha composted cow manure (approximately 15% organic matter); urea (N 46%), ammonium sulfate (N 20%), ammonium dihydrogen phosphate (N 11%, P_2_O_5_ 43%), calcium superphosphate (P_2_O_5_ 12%), fused calcium-magnesium phosphate (P_2_O_5_ 12%), and potassium chloride (K_2_O 60%) were applied as chemical NPK sources. (3) basic fertilizer and then 300 L/ha suspensions of strain ISOP5. (4) basic fertilizer and then 300 L/ha suspensions of strain ISP-1. (5) basic fertilizer and then both 300 L/ha suspensions of strain ISOP5 and 300 L/ha suspensions of strain ISP-1 (MB). 70% of the NPK fertilizers and all of the composted cow manure were applied at sowing time; the remaining 30% NPK fertilizers were applied 45 days after sowing. Bacterial inoculums were applied twice, with half of the volume applied at the sowing time and the other half applied at the pegging stage (in the middle of June). At sowing time, NPK fertilizers were sprinkled on soil surface. The soil was then plowed, and 13 or 14 ditches (about 5 cm deep) were dug in each plot. The bottom of these ditches was covered with composted cow manure, and bacteria inoculums were sprayed on manure, covered with 1cm of soil. Peanut seeds (*Arachis hypogaea* L. var. Ganhua 5, 1.2 kg/plot) were sowed by hand, and all ditches were filled with soil (Supplementary Figure 4). The remaining 30% NPK fertilizers were sprinkled on the soil around the peanut plants, and the additional bacterial inoculums were added on the soil of the peanut root zone. Each treatment contained four replicates (plots), and each plot was 66 m^2^ (10 m × 6.6 m). Plots were randomly arranged with a 20 cm trench between them. The site has been in peanut–radish crop rotation since 2000, and 100 kg/ha urea was applied to each plot at radish season.

### Collection of Yield Data and Sampling of Soil

Peanuts were harvested at physiological maturity in the 3rd week of August every year. The plants in the entire plot area were harvested to obtain yield. Seed samples were sun-dried, and peanut yields are reported at 15% moisture content. Five plants were randomly selected and sun-dried for 3–4 days to determine the biomass.

In August 2016, before harvesting, the soil samples near the root zone (approximately 5 cm away from the plant stem) from depths of 0–20 cm were collected using a core sampler (20 mm inner diameter). The soil from six randomly chosen points in each plot was mixed thoroughly to obtain a representative sample. The collected soil samples were sieved through a 2-mm mesh to remove large particles such as roots and stones. Then the soil samples were divided into two parts: one part was stored at −40°C until analysis, while the other was air dried at 22–30°C, sieved (< 2 mm), powdered in an agate mortar, and passed through a 0.1-mm sieve before being thoroughly mixed for further analysis.

### Analyses of Soil Physicochemical Properties

Soil pH was measured using a glass combination electrode (REX, shanghai, China) with a 1:1 ratio of soil to distilled water (Li et al., 2013). Soil cation exchange capacity (CEC) was measured using the ammonium acetate method as described by Pansu and Gautheyrou (2006). The dissolved total N (DTN) in the soil was extracted with 2M KCl and determined using a continuous flow analytical system (San + + System, Skalar, Holland). The OM and total nitrogen (TN) contents were determined according to the method outlined by Strickland and Sollins (1987). Available P (AP), available K (AK), total phosphorus (TP), and total potassium (TK) were measured using the methods described by Ding et al. (2017). Soil moisture content (MC) was determined by drying 5 g of each soil sample at 105°C until the sample reached a consistent weight.

### DNA Extraction and Amplicon Sequencing

Total community DNA was extracted from 3 soil samples collected from the treatments of *B. cepacia* ISOP5, *R. palustris* ISP-1, MB, and CK. For each sample, DNA was extracted from 0.5 g of soil using the FastDNA^®^ SPIN Kit for soil (MP Biomedicals, Santa Ana, CA, United States) according to the manufacturer’s protocol. The extracted DNA was quantified by a ND-1000 spectrophotometer (Thermo-Scientific, Wilmington, DE, United States) and stored at −20°C until further use. The bacterial 16S rRNA gene (380 bp) was paired-end sequenced (PE250) on an Illumina HiSeq platform (Novogene, Beijing, China) according to the standard protocols with the primer sets 515F and 806R (Peiffer et al., 2013). Subsequently, the sequences were deposited in the NCBI SRA database (accession no. PRJNA627297).

### Statistical Analyses

The effects of fertilization treatments on soil properties and the diversity and abundance of soil bacterial communities were assessed by one-way analysis of variance (ANOVA) with the least significance difference (LSD) *post hoc* test at *p* < 0.05, using SPSS 20.0 software (IBM, Armonk, NY, United States).

Bacterial α- and β-diversities were also analyzed. For α-diversity, the following indices were calculated using QIIME (V1.7.0) (Caporaso et al., 2010): observed species, Chao1, Shannon, Simpson, ACE, and Coverage. β-diversity patterns were assessed with non-metric multidimensional scaling (NMDS) plots using the Bray–Curtis dissimilarity matrix. Significant difference tests in the bacterial communities between different treatments were conducted using ANOSIM and non-parametric permutational multivariate analysis of variance (PERMANOVA, with the ‘adonis’ function) based on Bray-Curtis dissimilarities and 999 permutations. The β-diversity statistical analysis was processed by R version 3.6.3 using the package vegan and then plotted by Origin 2018. Heatmaps were performed and drawn with the R package pheatmap.

Software PICRUSt 1.1.4 (Langille et al., 2013) was used to predict metagenomic functional content from marker gene surveys and full genomes (Langille et al., 2013). Functional annotation was conducted by aligning sequencing reads against the Kyoto Encyclopedia of Genes and Genomes (KEGG) Orthology (KO). Briefly, the operational taxonomic units (OTUs) of 16S rRNA sequences were normalized in PICRUSt, and the KO profiles were calculated using the PICRUSt algorithm. Genes involved in soil microbial P-transformation in different treatments were searched for in the datasets based on previous publications (Dai et al., 2019). Genes related to nitrogen cycling (Supplementary Table 1) referenced to Sun and Badgley (2019) were searched for in the KEGG database. The “plspm” package in the R software (Sanchez, 2013) was used to explore the complex relationship amongst peanut yield values, soil properties, bacterial community diversity, bacterial composition, and bacterial function. GoF was used as an index of the average prediction for the entire model. The naive rule of GoF values is that the higher the value, the better. GoF values greater than 0.7 are considered “very good” within the partial least squares path modeling (PLS-PM) community. Figures were plotted using Origin 2018.

## Results

### Peanut Yield Over Five Consecutive Years

Data pertaining to the yield, shown in Table 1, revealed that MB had the highest yield over CK for four consecutive years, while conventional chemical fertilization had the lowest yield among the five treatments except for the year 2013. *B. cepacia* ISOP5, *R. palustris* ISP-1, and MB treatments recorded 13.9%, 6.5%, and 20.3% increases in peanut yield over CK in the fifth year, while the five-year average yield increased by 8.1%, 12.5%, and 19.5% over CK, respectively. Compared with conventional fertilizer treatment, *B. cepacia* ISOP5, *R. palustris* ISP-1, and MB treatments saw a five-year average yield increase rate of 11.1%, 15.7%, and 23%, respectively. Growth data in the fifth year, shown in Supplementary Table 2, indicate that growth parameters, such as plant height, significantly increased when *B. cepacia* ISOP5 and *R. palustris* ISP-1 inoculums were applied alone or in combination (MB). Furthermore, dual inoculation with *B. cepacia* ISOP5 and *R. palustris* ISP-1 continued to increase peanut yields by 8.86% to 40.46% throughout the five consecutive years (Table 1). There was no significant correlation between the peanut yield of each treatment and the rainfall (*R*^2^ = −0.2 ∼ 0.08, *P* > 0.05), maximum temperature (*R*^2^ = 0.308 ∼ 0.462, *P* > 0.05), or minimum temperature (*R*^2^ = −0.7 or −0.9, *P* > 0.05) during the peanut growing period (March to August each year), implying that rainfall and air temperature were not the main factors causing changes in yields over the years of 2012 ∼ 2016.

**TABLE 1:** Peanut yield for five consecutive years and yield increase rates with bacterial inoculum treatments compared with CK treatment.

	Conventional	CK	*B. cepacia* ISOP5		*R. palustris* ISP-1		MB	
	Yield (t/ha)	Yield (t/ha)	Yield (t/ha)	Changes relative to CK (%)	Yield (t/ha)	Changes relative to CK (%)	Yield (t/ha)	Changes relative to CK (%)
2012	5.38 ± 0.74b	5.93 ± 0.72ab	5.65 ± 0.54ab	–4.6	6.73 ± 0.64a	13.5	6.45 ± 0.4ab	8.9
2013	4.7 ± 0.11ab	3.65 ± 0.89b	4.64 ± 0.88ab	27.2	4.9 ± 0.8ab	34.4	5.12 ± 0.27a	40.5
2014	3.13 ± 0.17c	3.42 ± 0.11bc	3.64 ± 0.37ab	6.4	3.45 ± 0.26bc	0.8	4.06 ± 0.19a	18.5
2015	2.73 ± 0.17b	2.79 ± 0.21b	2.8 ± 0.17b	0.3	3.01 ± 0.3ab	7.8	3.19 ± 0.14a	14.3
2016	4.84 ± 0.19d	5.58 ± 0.21c	6.36 ± 0.28ab	13.9	5.95 ± 0.39bc	6.5	6.71 ± 0.23a	20.3
Average in 5 years	4.15 ± 0.24b	4.27 ± 0.29b	4.62 ± 0.31ab	8.1	4.81 ± 0.35ab	12.5	5.11 ± 0.02a	19.5

*Different lowercase letters after the data represent the significant difference among the 5 treatments at p < 0.05. Conventional: conventional chemical fertilization; CK: fertilized with basic fertilizer containing NPK fertilizers and composted cow manure; B. cepacia ISOP5: fertilized with basic fertilizer and then applied with suspension of strain B. cepacia ISOP5; R. palustris ISP-1: fertilized with basic fertilizer and then applied with suspension of strain R. palustris ISP-1; MB, fertilized with basic fertilizer and then applied with suspensions of B. cepacia ISOP5 and R. palustris ISP-1.*

### Changes in Nutrient Contents of Peanut and Soil Physicochemical Properties

The contents of N, P, K, and protein in peanut seeds, as well as soil physicochemical properties in the fifth year of harvest, are shown in Table 2. Inoculation of *B. cepacia* ISOP5 and *R. palustris* ISP-1 significantly promoted the absorption of N and increased the protein content of peanut seeds. Regarding soil physicochemical properties, the effects of *B. cepacia* ISOP5 and *R. palustris* ISP-1 were not significant, with only minor improvements seen in some nutrients compared with CK treatment, including improved DTN (12%), TN (10%), AP (33%), AK (12%), and OM (17%) in *B. cepacia* ISOP5 treatment; improved AP (25%) and AK (10%) in *R. palustris* ISP-1 treatment; and improved AP (13%) and AK (14%) in MB treatment. Although the input of chemical nutrients in conventional fertilizer was much higher than that in the CK and bacterial inoculum treatments, the residual nutrients in the soil were similar for conventional and CK treatments. Concentrations of AP, AK, and TP were lower when using conventional fertilizers than using CK after 5 years of fertilization and crop cultivation (Table 2).

**TABLE 2:** Nutrient contents in peanut seeds and soil physicochemical properties in the 5th year of harvest.

		Conventional	CK	*B. cepacia* ISOP5	*R. palustris* ISP-1	MB
Peanut seed nutrient contents	Protein%	23.85 ± 0.28ab	23.23 ± 0.86b	26.25 ± 2.76a	26.62 ± 1.78a	26.55 ± 1.41a
	N%	4.37 ± 0.05ab	4.26 ± 0.16b	4.81 ± 0.51a	4.88 ± 0.33a	4.86 ± 0.26a
	P%	0.42 ± 0.03a	0.41 ± 0.02a	0.42 ± 0.05a	0.41 ± 0.04a	0.42 ± 0.04a
	K%	0.7 ± 0.05a	0.79 ± 0.04a	0.76 ± 0.09a	0.76 ± 0.06a	0.76 ± 0.05a
Soil chemical properties	pH	4.92 ± 0.18a	4.67 ± 0.12b	4.84 ± 0.08ab	4.86 ± 0.11ab	4.81 ± 0.16ab
	EC(um/cm)^*a*^	45.7 ± 2.36c	62.23 ± 1.27ab	59.3 ± 8.73ab	57.39 ± 5.78b	67.9 ± 8.22a
	AP/(mg/kg)	23.13 ± 8.08a	30.97 ± 12.43a	41.06 ± 17.04a	38.71 ± 19.07a	34.99 ± 16.91a
	AK/(mg/kg)	188 ± 47.53b	207.24 ± 22.4ab	233 ± 28.89ab	228.94 ± 21.14ab	236.85 ± 10.81a
	DTN (mg/kg)	64.54 ± 3.07a	61.85 ± 8.33a	69.06 ± 5.95a	62.37 ± 10.47a	63.66 ± 2.06a
	OM (g/kg)	18.21 ± 0.5a	14.92 ± 1.13ab	17.38 ± 2.79a	15.87 ± 1.77ab	15.48 ± 2.29ab
	TN (mg/kg)	751.53 ± 18.61a	745.2 ± 38.48a	818.19 ± 31.36a	778.49 ± 73.58a	770.22 ± 38.32a
	TP (mg/kg)	483.02 ± 24.98a	487.88 ± 80.04a	495.73 ± 9.44a	489.41 ± 45.32a	506.69 ± 9.6a
	TK (%)	1.34 ± 0.03a	1.32 ± 0.09a	1.33 ± 0.06a	1.37 ± 0.07a	1.31 ± 0.09a

*^a^Electronic conductivity. Different lowercase letters represent the significant difference among the 5 treatments at p < 0.05. Conventional: conventional chemical fertilization; CK: fertilized with basic fertilizer containing NPK fertilizers and composted cow manure; B. cepacia ISOP5: fertilized with basic fertilizer and then applied with suspension of strain B. cepacia ISOP5; R. palustris ISP-1: fertilized with basic fertilizer and then applied with suspension of strain R. palustris ISP-1; MB, fertilized with basic fertilizer and then applied with suspensions of B. cepacia ISOP5 and R. palustris ISP-1.*

### Effects of Bacterial Inoculums on Soil Bacterial Communities

Soils under *B. cepacia* ISOP5, *R. palustris* ISP-1, MB, and CK treatments were selected, and amplification products of soil bacterial 16S rRNA gene were sequenced in order to determine the effects of the bacterial inoculums on soil bacterial communities. Based on the reading of 16S rRNA gene sequences, a total of 902,009 high quality sequences were obtained and clustered into 4,964 OTUs (97% identity). The bacterial alpha diversity indexes in the treated soils are shown in Table 3. These results showed that inoculation with *B. cepacia* ISOP5 or *R. palustris* ISP-1 for five years did not significantly change the bacterial diversity or richness in the soil, except that the observed species were significantly increased by *B. cepacia* ISOP5 inoculation (Table 3).

**TABLE 3:** Estimators of bacterial diversity, richness, and coverage in soil under different treatments.

Treatments	Observed species	Diversity^1^		Richness^2^		Coverage^3^
		Shannon	Simpson	Chao1	ACE	
CK	2515 ± 41.39b	9.18 ± 0.04a	0.996 ± 0a	2875.01 ± 237.45a	2960.92 ± 136.48a	0.989 ± 0.002a
*B. cepacia* ISOP5	2626 ± 47.15a	9.24 ± 0.06a	0.996 ± 0a	3006.7 ± 119.92a	3100.82 ± 42.66a	0.988 ± 0.002a
*R. palustris* ISP-1	2592 ± 43.39ab	9.26 ± 0.04a	0.996 ± 0a	2956.43 ± 197.5a	3033.81 ± 106.6a	0.988 ± 0.001a
MB	2599 ± 74.93ab	9.32 ± 0.15a	0.996 ± 0a	2882.55 ± 106.81a	2969.76 ± 107.52a	0.989 ± 0.002a

*Data are means ± standard deviation. Values followed by different letters are significantly different (p < 0.05) according to the Fisher LSD test. ^1^Based on the Shannon and Simpson diversity indices. A higher Shannon or lower Simpson index indicated higher bacterial community diversity. ^2^Based on the Chao1 and abundance-based coverage estimator (ACE) richness indices. ^3^Coverage: Good’s non-parametric coverage estimator. The higher the value, the lower the probability that the sequence was not measured in the sample. CK: fertilized with basic fertilizer containing NPK fertilizers and composted cow manure; B. cepacia ISOP5: fertilized with basic fertilizer and then applied with suspension of strain B. cepacia ISOP5; R. palustris ISP-1: fertilized with basic fertilizer and then applied with suspension of strain R. palustris ISP-1; MB, fertilized with basic fertilizer and then applied with suspensions of B. cepacia ISOP5 and R. palustris ISP-1.*

**FIGURE 1:**
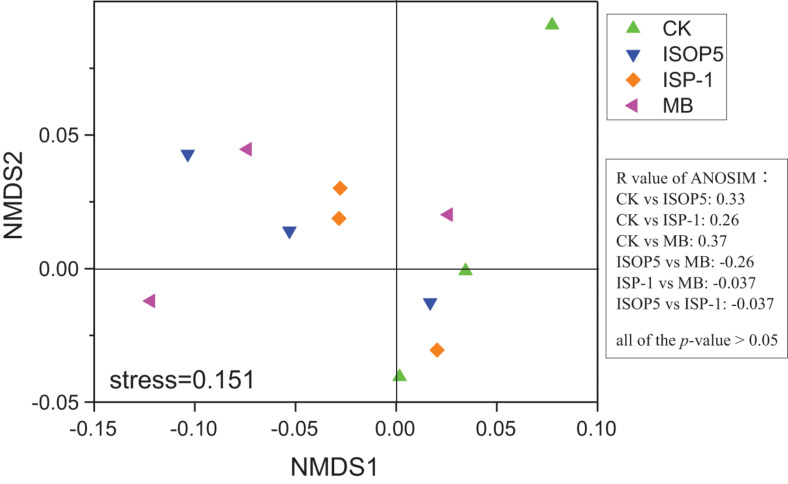
Non-metric multidimensional scaling (NMDS) plots of bacterial community composition in soils with and without bacteria inoculums. ANOSIM showed non-significant differences (*p* > 0.05) between treatments (*R* = degree of separation between test groups ranging from -1 to 1; *R* = 0, not different; *R* = 1, completely different; *p*-values were based on 9999 permutations). CK: fertilized with basic fertilizer containing NPK fertilizers and composted cow manure; *B. cepacia* ISOP5: fertilized with basic fertilizer and then applied with suspension of strain *B. cepacia* ISOP5; *R. palustris ISP-1*: fertilized with basic fertilizer and then applied with suspension of strain *R. palustris* ISP-1; MB: fertilized with basic fertilizer and then applied with suspensions of *B. cepacia* ISOP5 and *R. palustris* ISP-1.

**FIGURE 2:**
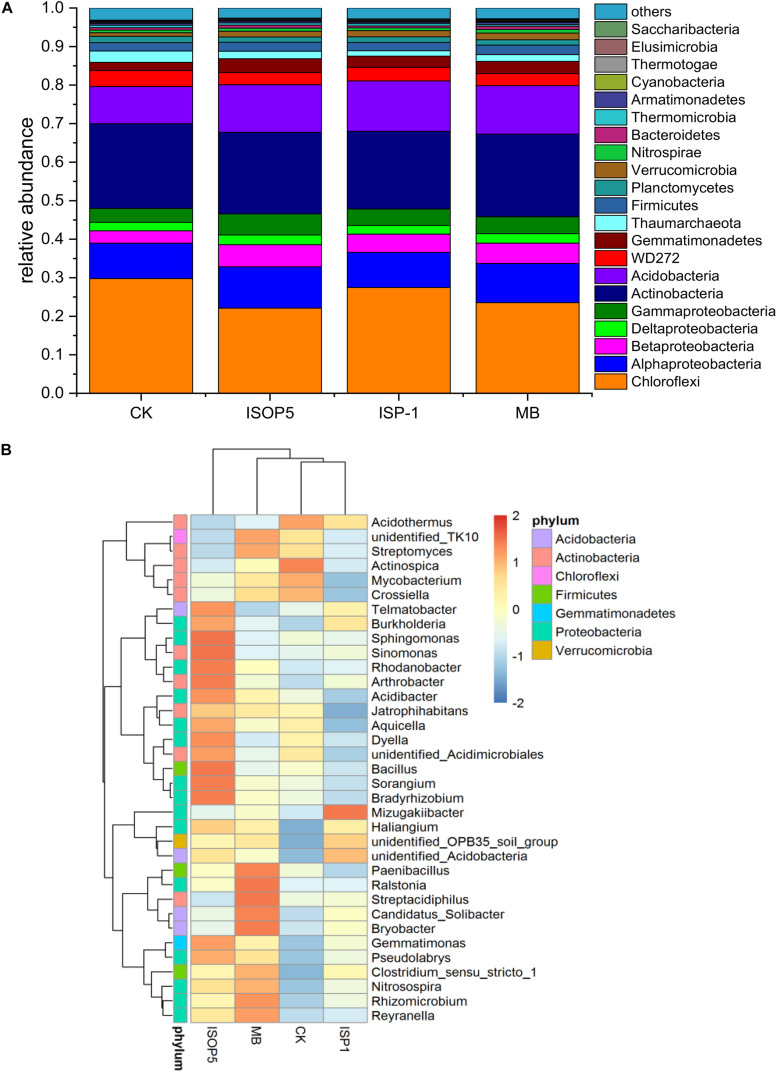
Relative abundance in the dominant bacterial phyla (proteobacterial classes) **(A)** and dominant genus **(B)** in the unique OTUs of each treatment. CK: fertilized with basic fertilizer containing NPK fertilizers and composted cow manure; *B. cepacia* ISOP5: fertilized with basic fertilizer and then applied with suspension of strain *B. cepacia* ISOP5; *R. palustris ISP-1*: fertilized with basic fertilizer and then applied with suspension of strain *R. palustris* ISP-1; MB: fertilized with basic fertilizer and then applied with suspensions of *B. cepacia* ISOP5 and *R. palustris* ISP-1.

NMDS based on Bray–Curtis distances was applied to analyze the overall structural variations of bacterial communities in different soils (Figure 1). NMDS ordination and ANOSIM analysis showed that there were no significant differences (*p* > 0.05) among treatments (Figure 1), and PERMANOVA analysis confirmed this result (R^2^ ranged from 0.16 to 0.28, and *p*-values ranged from 0.2 to 0.6). The relative abundances of different phyla/classes under different fertilizer treatments are shown in Figure 2. The soil bacteria communities were dominated by Chloroflexi, Proteobacteria, Actinobacteria, and Acidobacteria. In the corresponding communities, 25.7%, 21.3%, 21.2%, and 11.9% of the sequences were assigned to these four phyla, respectively. An independent-sample *t*-test identified Acidobacteria, Verrucomicrobia, and Bacteroidetes as significantly more abundant in soil treated by *B. cepacia* ISOP5 than that treated by CK, while only Verrucomicrobia had increased in *R. palustris* ISP-1 and MB groups (Supplementary Figure 5). Independent-sample *t*-test was also used to compare the differences at the genus level. We found that only *Chloroflexi_TK10* was significantly (*p* < 0.05) decreased by *B. cepacia* ISOP5 treatment, whereas *Verrucomicrobia_OPB35* had significantly (*p* < 0.05) increased in all of the soils inoculated with bacterial inoculums. These patterns suggest that inoculation with *B. cepacia* ISOP5 and/or *R. palustris* ISP-1 provided selective advantages for *Verrucomicrobia_OPB35*.

### Effects of Inoculums on Soil Microbial Functions With Regard to P Transformation

**FIGURE 3:**
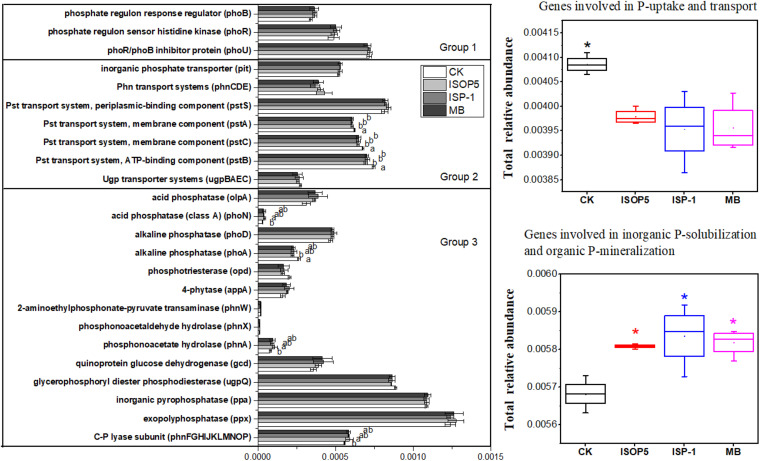
Relative abundance in representative genes responsible for microbial (1) P starvation response regulation, (2) P uptake and transport, and (3) inorganic P solubilization and organic P mineralization in soils treated with or without bacterial inoculums. The relative abundances of genes were calculated based on the annotated reads. Group 1: Genes coding for P starvation response regulation; Group 2: Genes coding for P uptake and transport; Group 3: Genes coding for inorganic P solubilization and organic P mineralization. Different lowercase letters and asterisks represent the significant effects of bacteria inoculums on the relative abundance of genes involved in P transformation at *p* < 0.05. Error bars are ± standard error. CK: fertilized with basic fertilizer containing NPK fertilizers and composted cow manure; *B. cepacia* ISOP5: fertilized with basic fertilizer and then applied with suspension of strain *B. cepacia* ISOP5; *R. palustris ISP-1*: fertilized with basic fertilizer and then applied with suspension of strain *R. palustris* ISP-1; MB: fertilized with basic fertilizer and then applied with suspensions of *B. cepacia* ISOP5 and *R. palustris* ISP-1.

Five years of inoculation with *B. cepacia* ISOP5 and/or *R. palustris* ISP-1 had no effect on the relative abundance of the genes involved in microbial P starvation response regulation (Figure 3). Nevertheless, the treatments decreased the total relative abundance of the genes involved in P uptake and transport by 2.62–3.24%, and increased the genes involved in inorganic P solubilization (IPS) and organic P mineralization (OPM) by 2.24–2.71% (*p* < 0.05) (Figure 3).

With regard to individual genes coding for P transport and uptake, *B. cepacia* ISOP5 and/or *R. palustris* ISP-1 inoculation decreased the relative abundance of the high-affinity phosphate-specific transporter (*pstACB*) (*p* < 0.05), whereas none of the bacterial inoculum treatments affected the relative abundance of the low-affinity inorganic phosphate transporter (*pit*) (*p* > 0.05) (Figure 3). As for individual genes coding for P solubilization and mineralization, *B. cepacia* ISOP5 increased the relative abundance of genes coding for acid phosphatase (*phoN*), phosphonoacetate hydrolase (*phnA*), and the C-P lyase subunit (*phnFGHIJKLMNOP*) (*p* < 0.05) but decreased the genes coding for alkaline phosphatase (*phoA*) (*p* < 0.05) (Figure 3). The soil treated with *R. palustris* ISP-1 and MB showed similar trends, but the changes were not significant.

### Effects of Inoculums on the N Metabolic Functions of Soil Microbes

**FIGURE 4:**
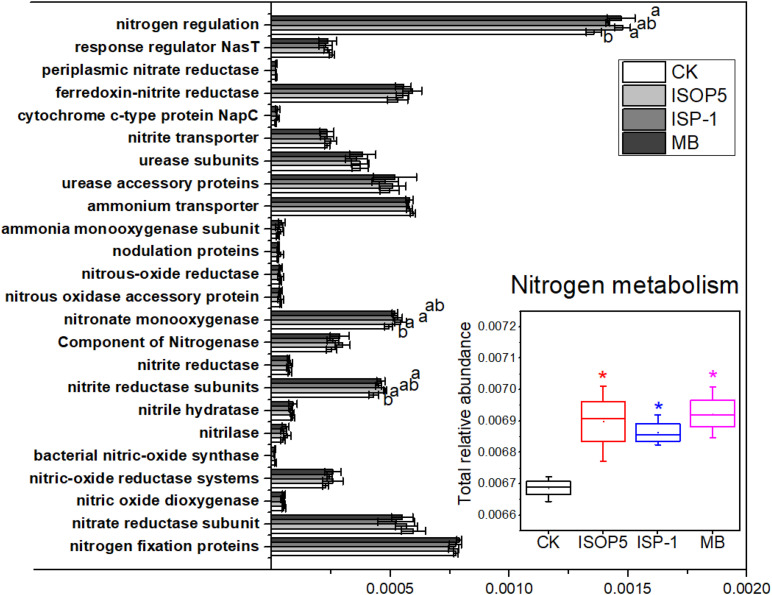
Major functional genes involved in the N cycle and total relative abundance of genes involved in N metabolism in soils treated with or without bacterial inoculums (relative abundance > 0.001%). Different lowercase letters and asterisks represent the significant effects of bacteria inoculums on the relative abundance of genes involved in the N cycle at *p* < 0.05. Error bars are ± standard error. CK: fertilized with basic fertilizer containing NPK fertilizers and composted cow manure; *B. cepacia* ISOP5: fertilized with basic fertilizer and then applied with suspension of strain *B. cepacia* ISOP5; *R. palustris ISP-1*: fertilized with basic fertilizer and then applied with suspension of strain *R. palustris* ISP-1; MB: fertilized with basic fertilizer and then applied with suspensions of *B. cepacia* ISOP5 and *R. palustris* ISP-1.

The total relative abundance of the genes associated with nitrogen metabolism was significantly increased by bacterial inoculums. Consistent with taxonomic findings, there was no significant change in most of the functional genes involved in N cycling, except for genes involved in nitrogen regulation, nitrite reductase subunits, and nitronate monooxygenase (*p* < 0.05) (Figure 4). Nitrite reductase, a key enzyme in the natural N cycle, can degrade nitrite into NO or NH_3_. Its increase thus implies higher denitrifying activity in the treated soils. Furthermore, genes coding for N fixation proteins were not significantly affected by bacterial inoculums (Figure 4). However, soils inoculated with *B. cepacia* ISOP5 had improved DTN (12%) and TN (10%) compared with soils treated by CK (Table 2).

## Discussion

### Peanut Yield Increased With Lower Chemical Fertilizer Input; Combined Application of *B. cepacia* ISOP5 and *R. palustris* ISP-1 Is More Effective Than Separate Application for Increasing Yield

The use of PSB to obtain higher yields has been reported as a promising approach for various crops (Valetti et al., 2018). PSB is capable of solubilizing (e.g., by producing organic acids and reducing soil pH) and mineralizing complex P compounds (e.g., through the production of phosphatase), thus making inorganic and organic P sources in soil into a state that can be absorbed by plants (Sharma et al., 2013; Rezakhani et al., 2019). PNSB are a group of dinitrogen (N_2_)-fixing (Ludden and Roberts, 2002) and polyphosphate-accumulating organisms (Liang et al., 2010). The yield-increasing effect of PSB and PNSB has been previously reported by Jaybhay et al. (2017) and Wu et al. (2013). Nevertheless, here, we report for the first time the yield-increasing effect on a long-term basis from inoculation with PSB *B. cepacia* ISOP5 and PNSB *R. palustris* ISP-1. Compared with conventional fertilizer treatment, *B. cepacia* ISOP5, *R. palustris* ISP-1, and MB increased the five-year average yield by 11.1%, 15.7%, and 23%, respectively (Table 1), while reducing 87.1 kg N ha^–1^, 20 kg P_2_O_5_ ha^–1^, and 65 kg K_2_O ha^–1^ of chemical fertilizer input. Furthermore, the residual chemical nutrients in the soil of the conventional fertilizer treated plots were similar to those treated with CK (Table 2) after 5 years of fertilization and crop cultivation. This result indicated that the loss of fertilizer nutrients in soil under conventional fertilizer treatment was more serious, and the addition of composted cow manure may increase the ability of soil to fix chemical nutrients. Higher yields in the bacterial inoculum amended plots might be due mostly to the positive effects of the bacterial inoculums on peanut N uptake through improved N fertilizer use efficiency in soils. The composted cow manure might also play an important role, as the five-year average yield recorded a 3% increase in CK treatment compared with conventional fertilizer treatment (Table 1). This result was consistent with those of previous studies which reported that chemical fertilizer reduction and organic fertilizer addition can improve soil quality, increase SOM, maintain soil productivity, and increase vegetable or grain yields (Wang et al., 2020; Han et al., 2021).

In addition, we found that the combination of *B. cepacia* ISOP5 and *R. palustris* ISP-1 treatment is more effective in enhancing peanut yield than separate application (Table 1). The dual inoculation of *B. cepacia* ISOP5 and *R. palustris* ISP-1 significantly increased the dry weight of peanut plants (Supplementary Table 2). Peanut plant height and yield increase can be attributed to nutrient (such as N, AP, and AK) availability due to *B. cepacia* ISOP5 and *R. palustris* ISP-1 inoculation (Table 2), which causes the soil to become biologically and physico-chemically rich, thereby promoting plant growth. In addition, the increase may also be due to the fact that PSB may produce various organic compounds in the plant rhizosphere such as IAA, cytokinin, and gibberellin (Rafique et al., 2017).

### Inoculation of ISOP5 and/or ISP-1 Promoted the Growth of Peanut Plant and Increased Nitrogen and Protein Concentrations in Peanut Seeds

Inoculation with PSB or PNSB can promote plant growth and result in higher crop yields (Wu et al., 2013; Valetti et al., 2018). *Burkholderia cepacia* JFW16, which belongs to the genus *Burkholderia* and the β-proteobacteria class, has the capacity to promote growth and enhance photosynthesis in inoculated plants (Zhou et al., 2018). Moreover, in a previous study, *B. tropica*-inoculated plants showed a consistent increase in both the number and weight of tomato fruits when compared to uninoculated controls (Bernabeu et al., 2015). The bacterium *R. palustris* also promotes plant growth (Wong et al., 2014; Xu et al., 2018). Inoculating maize plants with PSB *Lysinibacillus fusiformis* strain 31MZR can enhance N and P uptake (Rafique et al., 2017). In this study, after five years of repeated inoculation, both *B. cepacia* ISOP5 and *R. palustris* ISP-1 significantly promoted the growth of peanut plants (Supplementary Table 2) and increased the nitrogen and protein content in peanut seeds (Table 2). This was consistent with the previous results and further indicated that *B. cepacia* ISOP5 and *R. palustris* ISP-1 could promote nitrogen absorption in peanut plants. Soil inoculated with *B. cepacia* ISOP5 had improved DTN (12%) and TN (10%) compared with CK-treated soil, while DTN only improved 1% and 3% and TN only improved 4% and 3%, respectively, in soil treated with *R. palustris* ISP-1 and MB. On the other hand, the N content in peanut seeds (Table 2) and the growth parameters such as the dry weight of plants (Supplementary Table 2) were higher with *R. palustris* ISP-1 and MB treatments than *B. cepacia* ISOP5 treatment. Therefore, we speculate that the plants in *R. palustris* ISP-1 and MB treatments may have stronger ability to absorb N than plants in *B. cepacia* ISOP5 treatment, resulting in less residual DTN and TN in soil at harvest time.

### Few Bacterial Groups Were Increased by Bacterial Inoculums

In this study, after inoculation with *B. cepacia* ISOP5 and *R. palustris* ISP-1 for five years, bacterial diversity and richness in the soil were not significantly affected. Similarly, Xu et al. (2018) reported that soil bacterial diversity was not significantly influenced by *R. palustris* inoculation with or without chemical fertilization. However, Xu et al. (2016) also reported that *R. palustris* inoculated via foliar spray increased the abundance of the phyla Acidobacteria and Actinobacteria, which is different from the results of this study. This may be due to differences in soil types, application modes, and crops used. In addition, agricultural soil contains highly diverse indigenous microorganisms, which can hinder the colonization of soil by exogenous bacteria inoculants. Furthermore, the composition, abundance, and activity of bacterial populations are regulated by a variety of biotic and abiotic factors such as climate, seed properties, plant characteristics, soil management, soil properties, as well as the presence of grazers and other members of microbial communities (Rilling et al., 2019). These factors may have differently affected the inoculum bacteria in different studies.

Three phyla (Acidobacteria, Verrucomicrobia, and Bacteroidetes) were significantly increased with *B. cepacia* ISOP5 application, while in *R. palustris* ISP-1 and MB treated soils, only the phylum Verrucomicrobia was significantly increased (Supplementary Figure 5). The community structure and abundance of Verrucomicrobia are extremely sensitive to changes of chemical factors linked to soil fertility (Navarrete et al., 2015). Previous studies have shown that Verrucomicrobia members are degraders of recalcitrant organic matter (Li Y. et al., 2017), which may play an important role in effectively degrading organic matter and increasing the level of OM content in soils inoculated with bacterial inoculums (Table 2).

The use of bacterial inoculants, either alone or in combination with other products, would reduce the amount of chemical fertilizer required or allow plants to use the phosphate already present in the soil (Lobo et al., 2019). Notably, short-term fertilization with bacterial inoculants has been found to influence soil microbial community composition (Xu et al., 2016), but significant changes in soil microbial community structure and diversity in the long term were not observed in this study (Table 3). This indicates a different response of the bacterial community in relation to short- and long-term additions of bacterial inoculums. The relative abundance of *Burkholderia* and *Rhodopseudomonas* were not significantly different among our treatments (Supplementary Figure 6). One possible explanation for this is that soil samples were collected at harvest time, months after inoculation, which was a lengthy period for *B. cepacia* ISOP5 and *R. palustris* ISP-1 to maintain large concentrations and high levels of activity. Therefore, further investigation based on time-dependent series would be helpful in understanding the influence of *B. cepacia* ISOP5 and *R. palustris* ISP-1 inoculation on soil microorganisms.

### Bacteria Inoculums Upregulated Genes Involved in IPS, OPM, and N Metabolism of Soil Microbes

Seed inoculation with PSB is an effective technique to alleviate the shortage of phosphorus (Lobo et al., 2019). Inoculation with *B. cepacia* ISOP5 and *R. palustris* ISP-1 over five years increased the microbial genes involved in IPS and OPM, but reduced the genes involved in P uptake and transport (Figure 3). Genes involved in IPS and OPM enable microorganisms to release organic anions to solubilize inorganic P or release enzymes to mineralize organic P. Genes coding for P uptake and transport systems enable microorganisms to efficiently utilize P and immobilize P in their biomass and may cause them to compete for the available P with plants in agro-ecosystems (Richardson and Simpson, 2011). The concentration of AP in bacteria inoculum treated soil increased by 12.98–32.58% when compared to that in CK-treated soil (Table 2). Genes involved in IPS and OPM may have played an important role in this phenomenon.

As one of the genes involved in microbial IPS, the *gcd* gene regulates the solubilization of unavailable mineral P, including Ca phosphates, hydroxyapatite, and some rock phosphates. The mineralization of soil organic P is mainly attributed to the genes coding for phosphatases, such as *phoA* and *phoD* genes (Kageyama et al., 2011; Dai et al., 2019). Our study revealed that inoculation with PSB *B. cepacia* ISOP5 did not increase the abundance of the *gcd* gene when compared to CK (Figure 3). However, significant interactive effects of *B. cepacia* ISOP5 were observed in the relative abundances of some specific genes coding for acid phosphatase (*phoN*), phosphonoacetate hydrolase (*phnA*), and C-P lyase subunits (Figure 3). At the same time, the concentration of AP in *B. cepacia* ISOP5-treated soil increased by 32.58% when compared to that in CK-treated soil (Table 2). These results indicate that the increase in soil phosphorus content may mainly be due to the increase in the soil OPM, rather than microbial IPS. The soil treated with *R. palustris* ISP-1 and MB showed similar trends.

Abundance changes in N cycling organisms indicate shifts in the overall N cycling process as well as shifts within functional groups (Sun and Badgley, 2019). In this study, inoculation with *B. cepacia* ISOP5 and *R. palustris* ISP-1 over five years significantly increased the total relative abundance of genes involved in N metabolism (Figure 4), indicating that N metabolism in soil inoculated with *B. cepacia* ISOP5 and/or *R. palustris* ISP-1 was more active. This, in turn, implies that *B. cepacia* ISOP5 and *R. palustris* ISP-1 may actively coordinate the soil microbial community to regulate soil nutrients so as to achieve the best plant growth. PNSB can utilize various substrates such as organic matter, nitrate, ammonia, and sulfide to metabolize under light or oxygenated conditions, because it has a variety of energy and substance metabolism pathways (Lu et al., 2011). A previous study reported that PNSB can remove nitrate from nitrogen-contaminated lakes or aquarium water and has great potential for treating a variety of nitrogen-contaminated wastewater (Yang et al., 2019). Furthermore, PNSB inoculation did not affect phototrophic and heterotrophic nitrogenase activities (acetylene-reducing activity) in the soil (Harada et al., 2005). These previous studies indicate that PNSB has the ability to regulate soil nitrogen metabolism.

Genes coding for N fixation proteins were not significantly affected by our bacterial inoculums (Figure 4). However, soil inoculated with *B. cepacia* ISOP5 had improved DTN (12%) and TN (10%) compared with CK-treated soil, while in the soil treated with *R. palustris* ISP-1 and MB, DTN improved by 1% and 3%, and TN improved by 4% and 3%, respectively (Table 2). Therefore, we assumed that the beneficial effects of *B. cepacia* ISOP5 and/or *R. palustris* ISP-1 inoculation on soil nitrogen content may not be due to biological N_2_ fixation. Instead, improved soil fertility and increased ability of soil to retain nitrogen in bacterial inoculum amended plots might be the reasons. Other unidentified function changes, such as the significantly increased relative abundance of genes coding for nitronate monooxygenase and nitrogen regulation proteins or components (Figure 4), may also play an important role in the increase of soil N content and yield of peanuts. In order to further illuminate the role of *B. cepacia* ISOP5 and *R. palustris* ISP-1 in promoting plant growth and increasing crop yield, future studies should examine the dynamic changes in soil nutrients and microbial communities after bacterial inoculum application.

### Direct Effects of Bacteria Inoculums Contributed the Most to Improved Peanut Yield

**FIGURE 5:**
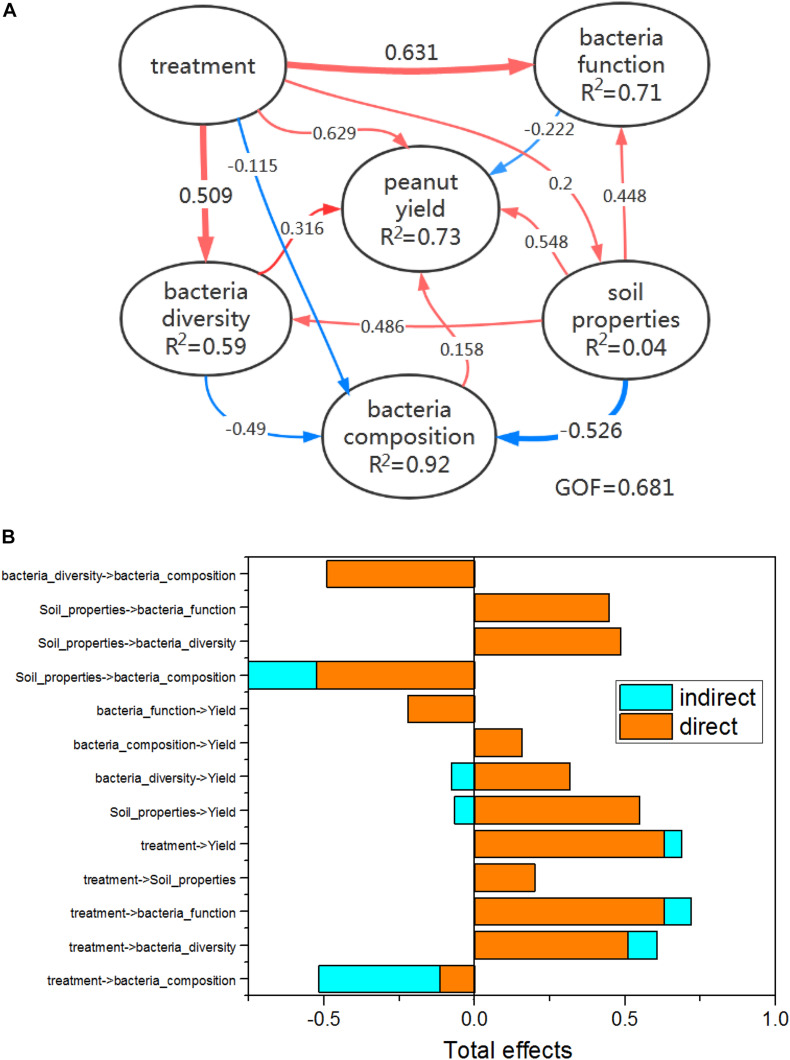
**(A)** Direct and indirect effects of treatments, soil properties (pH, AP, OM, DTN, and TN), bacteria diversity, bacteria composition, and bacteria functions (abundance of genes involved in P and N metabolism) on peanut yield were shown using PLS-PM. Path coefficients (i.e., direct effects) are written on arrows, and significant coefficients are shown in bold (*p* < 0.05). Arrows with positive and negative coefficients are shown in red and blue, respectively. *R*^2^ values represent the variance of dependent variables explained by the inner model. GOF denotes the goodness of fit index. **(B)** Standardized total effects (i.e., direct plus indirect effects) were calculated using PLS-PM.

PLS-PM analysis was employed to describe the specific causality among bacterial inoculums, bacterial communities, soil properties, and peanut yield (Figure 5). The results indicate that the direct effects (path coefficient = 0.629) of bacterial inoculums on peanut yield variation were greater than those of soil properties and bacterial communities, although all of their contributions were not significant (Figure 5B). Ramakrishna et al. (2019) reported that PGPB usually promotes plant growth through two mechanisms: 1) direct facilitation of resource acquisition and modulation of plant hormone levels, or 2) indirect inhibition of the various pathogens that hamper the growth and development of plants. Furthermore, PGPB have been found to produce indole acetic acid (IAA), siderophores, or biofilms, making phosphorus available to plants by phosphate solubilization (Ramakrishna et al., 2020). *R. palustris* ISP-1 can secrete EPS, which is an essential component of bacterial biofilm and can exert various biological functions such as adhesion to host surface, colonization, stress resistance, and modulation of host immunity against bacterial invasion (Zhai et al., 2021). Therefore, we speculate that the biomass of *B. cepacia* ISOP5 and *R. palustris* ISP-1 inoculums, or their metabolites, may be the main reason for the increase in peanut yield. The following factors likely play indirect roles in promoting peanut growth and improving peanut yield: the minor effects of the bacterial inoculums on soil bacterial diversity (Table 3), the increase in the bacteria phyla, Acidobacteria, Verrucomicrobia, and Bacteroidetes (Supplementary Figure 5), and the significant effects of the bacterial inoculums on genes involved in soil microbial P and N metabolism (Figures 3, 4). This is a conjecture based only on the analysis of the physical and chemical properties of the soil and the bacterial communities in the soil samples, which were collected along with the peanut harvest. PGPB can alter the structure of a resident microbial community, but these alterations depend on PGPB’s interactions with the resident microbial community and have temporary, spatially limited, and transient effects (Qiao et al., 2017; Ramakrishna et al., 2019). Therefore, if we can dynamically monitor the survival of bacterial inoculums in soil as well as soil nutrient change after inoculation, we can explain to a greater extent the reason why *B. cepacia* ISOP5 and *R. palustris* ISP-1 promote peanut yield.

## Conclusion

This study showed that the direct application of *B. cepacia* ISOP5 and *R. palustris* ISP-1 increased peanut yield, resulted in good crop growth, promoted crop absorption of N, and increased protein content in peanut seeds compared to chemical fertilizer without bacterial addition. Three reasons may explain the increase in peanut yield. First, *B. cepacia* ISOP5 and *R. palustris* ISP-1 bacterial inoculums enriched the soil with more available P and N. Second, inoculation of *B. cepacia* ISOP5 and *R. palustris* ISP-1 significantly increased the amount of *Verrucomicrobia_OPB35*, which are degraders of recalcitrant organic matter in soil. Third, these bacterial inoculums affected the soil microbial functions involved in P transformation and N metabolism by decreasing total relative abundance of the genes involved in P uptake and transport, increasing the abundance of genes involved in inorganic P solubilization and organic P mineralization, and increasing the abundance of genes involved in N metabolism. PLS-PM analysis showed that the direct effect of bacterial inoculants on peanut yield was the most important contributor to the yield increase. Therefore, *B. cepacia* ISOP5 and *R. palustris* ISP-1 are potential biological fertilizers that may improve soil fertility and microbial metabolism activities, ultimately increasing crop yields.

## Data Availability Statement

The data presented in the study are deposited in the NCBI sequence read archive (SRA) repository, accession number (PRJNA627297).

## Author Contributions

YW and Xg-L initiated the project. SP, QH, CQ and PW performed the experiments, Xl-L provided the meteorological data of monthly rainfall and air temperature during 2012–2016. SP analyzed the data and wrote the manuscript. All authors read and approved the final manuscript.

## Conflict of Interest

The authors declare that the research was conducted in the absence of any commercial or financial relationships that could be construed as a potential conflict of interest.
