# Supplementary material for: The Long-Term Effects of Using Phosphate-Solubilizing Bacteria and Photosynthetic Bacteria as Biofertilizers on Peanut Yield and Soil Bacteria Community
Source: Front Microbiol. 2021 Jul 16;12:693535. doi: 10.3389/fmicb.2021.693535 (PMC8322663; doi:10.3389/fmicb.2021.693535)
Supplement: Supplementary file 1 [file Data_Sheet_1.docx]

**Supporting Information**

**for**

**The long-term effects of using phosphate-solubilizing bacteria and photosynthetic bacteria as biofertilizers on peanut yield and soil bacteria community**

(A)


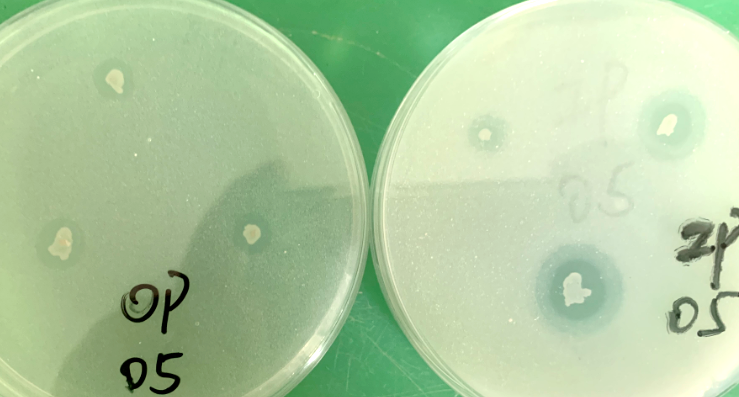


(B)


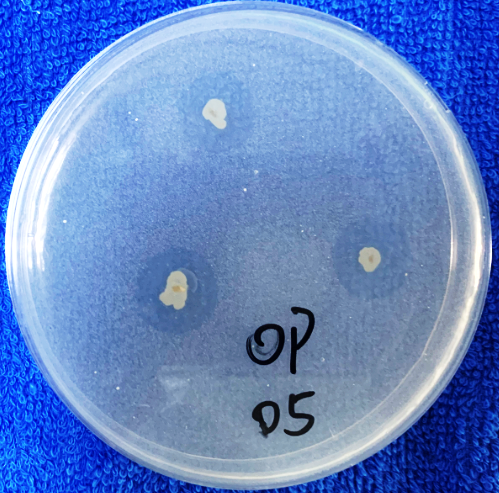

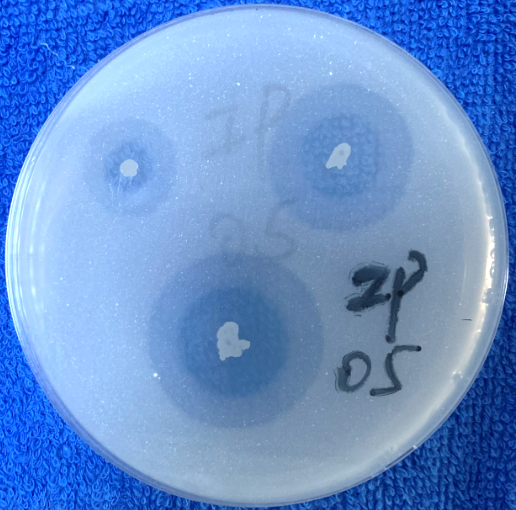


**Figure S1.** The diameter of *B. cepacia* ISOP5 solubilization halo on plate of tricalcium phosphate medium (IP) and lecithin medium (OP) after incubated for 48h (A) and 120h (B).

**Figure S2.** (A) Monthly rainfall of the experimental site during 2012 ~ 2016 and (B) the total rainfall during the peanut growing period (March to August each year).

**Figure S3.** The maximum and minimum air temperature during the experiment (2012-2016).


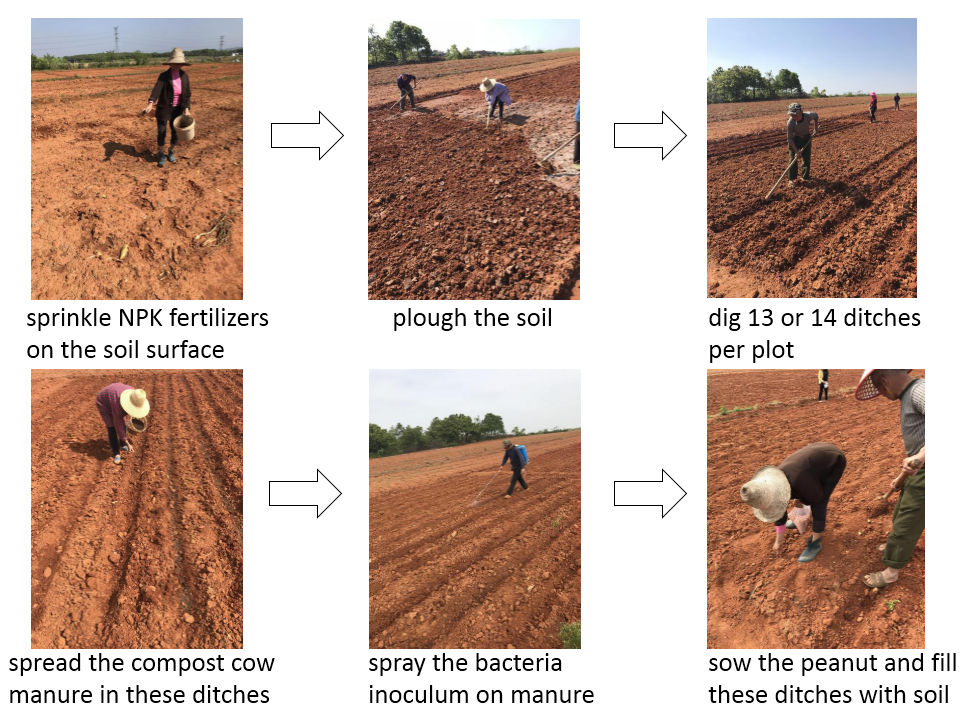


**Figure S4.** The fertilization method at sowing time


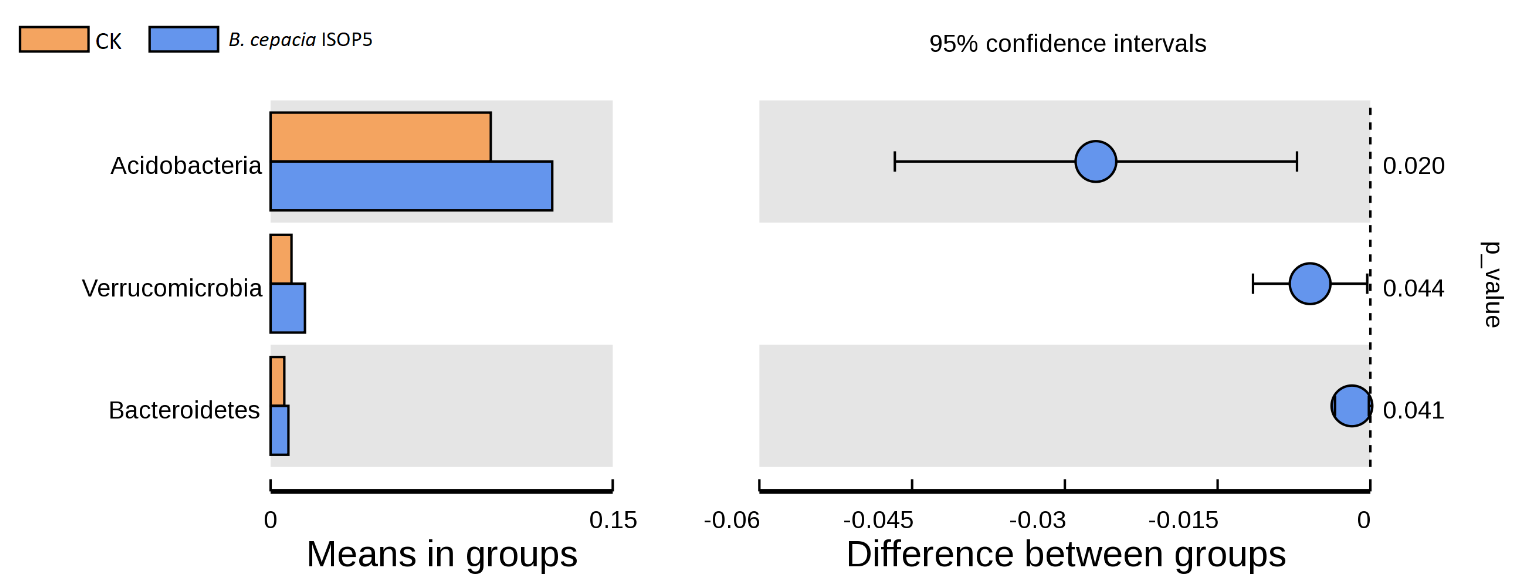


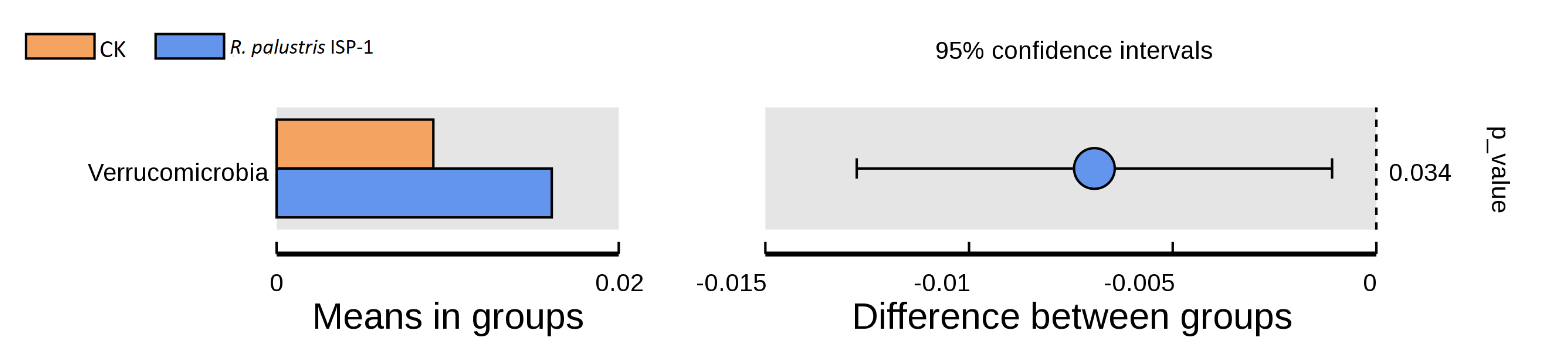


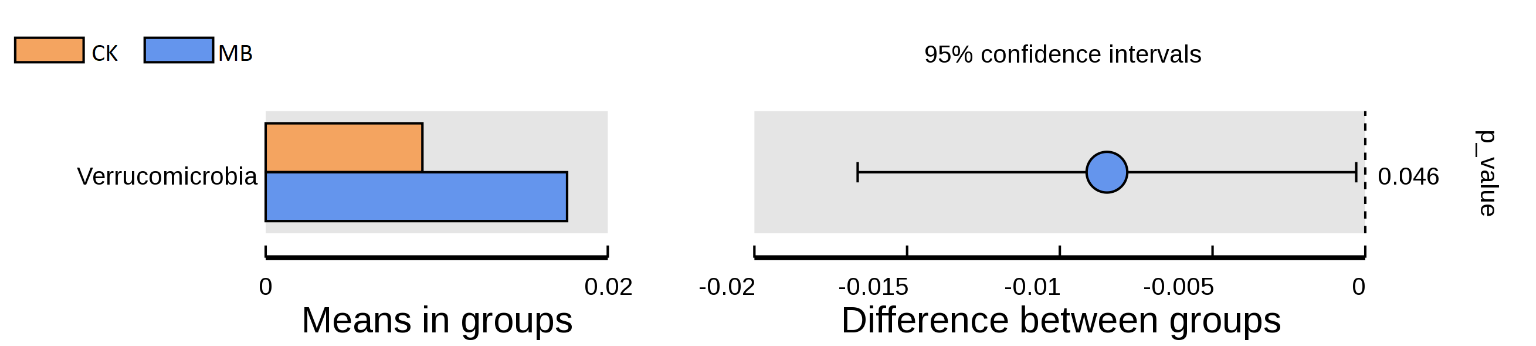


**Fig S5.** Differences in the abundances of bacteria phylum between CK and bacterial inoculum treatments. *P* values were calculated using Student’s t test (*P* < 0.05)

**Fig S6.** Relative abundance of the genus of Burkholderia and Rhodopseudomonas in the soils of each treatment based on amplicon sequencing data.

**Table S1.** The KO number, KEGG descriptions, and classification of the genes responsible for microbial nitrogen metabolism and regulatory or transport.

| KO number | Classification | KEGG_Description |
| --- | --- | --- |
| K00360 | nitrate reductase | nitrate reductase (NADH) [EC:1.7.1.1] |
| K00369 | nitrate reductase | nitrate reductase [EC:1.7.99.4] |
| K00370 | nitrate reductase subunit | nitrate reductase 1, alpha subunit [EC:1.7.99.4] |
| K00371 | nitrate reductase subunit | nitrate reductase 1, beta subunit [EC:1.7.99.4] |
| K00373 | nitrate reductase subunit | nitrate reductase 1, delta subunit [EC:1.7.99.4] |
| K00374 | nitrate reductase subunit | nitrate reductase 1, gamma subunit [EC:1.7.99.4] |
| K00372 | nitrate reductase subunit | nitrate reductase catalytic subunit [EC:1.7.99.4] |
| K05916 | nitric oxide dioxygenase | nitric oxide dioxygenase [EC:1.14.12.17] |
| K12265 | nitric-oxide reductase systems | nitric oxide reductase FlRd-NAD(+) reductase [EC:1.18.1.-] |
| K04747 | nitric-oxide reductase systems | nitric oxide reductase NorF protein;nitric-oxide reductase NorF protein [EC:1.7.99.7] |
| K04748 | nitric-oxide reductase systems | nitric oxide reductase NorQ protein;nitric-oxide reductase NorQ protein [EC:1.7.99.7] |
| K04561 | nitric-oxide reductase systems | nitric oxide reductase, cytochrome b-containing subunit I [EC:1.7.2.5];nitric-oxide reductase, cytochrome b-containing subunit I [EC:1.7.99.7] |
| K02448 | nitric-oxide reductase systems | nitric-oxide reductase NorD protein [EC:1.7.99.7];nitric oxide reductase NorD protein |
| K02164 | nitric-oxide reductase systems | nitric-oxide reductase NorE protein [EC:1.7.99.7];nitric oxide reductase NorE protein |
| K02305 | nitric-oxide reductase systems | nitric-oxide reductase, cytochrome c-containing subunit II [EC:1.7.99.7];nitric oxide reductase, cytochrome c-containing subunit II |
| K00491 | bacterial nitric-oxide synthase | nitric-oxide synthase, bacterial [EC:1.14.13.39] |
| K01501 | nitrilase | nitrilase [EC:3.5.5.1] |
| K01721 | nitrile hydratase | nitrile hydratase [EC:4.2.1.84] |
| K00362 | nitrite reductase subunit | nitrite reductase (NAD(P)H) large subunit [EC:1.7.1.4] |
| K00363 | nitrite reductase subunit | nitrite reductase (NAD(P)H) small subunit [EC:1.7.1.4] |
| K00368 | nitrite reductase | nitrite reductase (NO-forming) [EC:1.7.2.1] |
| K02598 | nitrite transporter | nitrite transporter NirC |
| K02585 | nitrogen fixation protein | nitrogen fixation protein NifB |
| K02593 | nitrogen fixation protein | nitrogen fixation protein NifT |
| K04488 | nitrogen fixation protein | nitrogen fixation protein NifU and related proteins |
| K02595 | nitrogen fixation protein | nitrogen fixation protein NifW |
| K02596 | nitrogen fixation protein | nitrogen fixation protein NifX |
| K02597 | nitrogen fixation protein | nitrogen fixation protein NifZ |
| K10851 | nitrogen regulation | nitrogen regulatory protein A |
| K02589 | nitrogen regulation | nitrogen regulatory protein PII 1 |
| K04751 | nitrogen regulation | nitrogen regulatory protein P-II 1 |
| K02590 | nitrogen regulation | nitrogen regulatory protein PII 2 |
| K04752 | nitrogen regulation | nitrogen regulatory protein P-II 2 |
| K07708 | nitrogen regulation | two-component system, NtrC family, nitrogen regulation sensor histidine kinase GlnL [EC:2.7.13.3] |
| K07712 | nitrogen regulation | two-component system, NtrC family, nitrogen regulation response regulator GlnG |
| K13598 | nitrogen regulation | two-component system, NtrC family, nitrogen regulation sensor histidine kinase NtrY [EC:2.7.13.3] |
| K13599 | nitrogen regulation | two-component system, NtrC family, nitrogen regulation response regulator NtrX |
| K02806 | nitrogen regulation | PTS system, nitrogen regulatory IIA component [EC:2.7.1.69] |
| K00536 | nitrogenase (flavodoxin) | nitrogenase (flavodoxin) [EC:1.19.6.1] |
| K00531 | nitrogenase | nitrogenase [EC:1.18.6.1] |
| K02588 | Component of Nitrogenase | nitrogenase iron protein NifH [EC:1.18.6.1] |
| K02587 | Component of Nitrogenase | nitrogenase molybdenum-cofactor synthesis protein NifE |
| K02586 | Component of Nitrogenase | nitrogenase molybdenum-iron protein alpha chain [EC:1.18.6.1] |
| K02591 | Component of Nitrogenase | nitrogenase molybdenum-iron protein beta chain [EC:1.18.6.1] |
| K02592 | Component of Nitrogenase | nitrogenase molybdenum-iron protein NifN |
| K00459 | nitronate monooxygenase | nitronate monooxygenase [EC:1.13.12.16] |
| K10679 | nitroreductase/dihydropteridine reductase | nitroreductase / dihydropteridine reductase [EC:1.-.-.- 1.5.1.34] |
| K10678 | nitroreductase | nitroreductase [EC:1.-.-.-] |
| K07218 | nitrous oxidase accessory protein | nitrous oxidase accessory protein |
| K00376 | nitrous-oxide reductase | nitrous-oxide reductase [EC:1.7.99.6];nitrous-oxide reductase [EC:1.7.2.4] |
| K14658 | nodulation proteins | nodulation protein A [EC:2.3.1.-] |
| K14660 | nodulation proteins | nodulation protein E [EC:2.3.1.-] |
| K14661 | nodulation proteins | nodulation protein F [EC:2.3.1.-] |
| K10944 | ammonia monooxygenase subunit | ammonia monooxygenase subunit A [EC:1.13.12.-] |
| K10945 | ammonia monooxygenase subunit | ammonia monooxygenase subunit B [EC:1.13.12.-] |
| K10946 | ammonia monooxygenase subunit | ammonia monooxygenase subunit C [EC:1.13.12.-] |
| K03320 | ammonium transporter | ammonium transporter, Amt family |
| K03189 | urease accessory proteins | urease accessory protein |
| K03188 | urease accessory proteins | urease accessory protein |
| K03190 | urease accessory proteins | urease accessory protein |
| K03187 | urease accessory proteins | urease accessory protein |
| K03192 | urease accessory proteins | urease accessory protein |
| K01428 | urease subunits | urease alpha subunit [EC:3.5.1.5];urease subunit alpha [EC:3.5.1.5] |
| K01430 | urease subunits | urease gamma subunit [EC:3.5.1.5];urease subunit gamma [EC:3.5.1.5] |
| K01429 | urease subunits | urease subunit beta [EC:3.5.1.5];urease beta subunit [EC:3.5.1.5] |
| K14048 | urease subunits | urease subunit gamma/beta [EC:3.5.1.5] |
| K10850 | nitrite transporter | MFS transporter, NNP family, putative nitrate transporter |
| K02575 | nitrite transporter | MFS transporter, NNP family, nitrate/nitrite transporter |
| K02569 | cytochrome c-type protein NapC | cytochrome c-type protein NapC |
| K00366 | ferredoxin-nitrite reductase | ferredoxin-nitrite reductase [EC:1.7.7.1] |
| K00367 | ferredoxin-nitrite reductase | ferredoxin-nitrate reductase [EC:1.7.7.2] |
| K00366 | ferredoxin-nitrite reductase | ferredoxin-nitrite reductase [EC:1.7.7.1] |
| K02567 | periplasmic nitrate reductase | periplasmic nitrate reductase NapA [EC:1.7.99.4] |
| K02570 | periplasmic nitrate reductase | periplasmic nitrate reductase NapD |
| K02571 | periplasmic nitrate reductase | periplasmic nitrate reductase NapE |
| K07183 | response regulator NasT | response regulator NasT |

**Table S2**. Effect of different fertilizers on growth parameters of peanut plants and peanut seeds after five years of continuous fertilization.

|  | Conventional | CK | *B. cepacia* ISOP5 | *R. palustris* ISP-1 | MB |
| --- | --- | --- | --- | --- | --- |
| Fresh weight of overground part from 5 plants (g) | 152.39±24.41c | 169.89±15.1bc | 179.53±23.77bc | 173.24±16.14bc | 252.27±16.49a |
| Dry weight of overground part from 5 plants (g) | 49.05±8.02c | 60.73±9.7bc | 58.86±8.36bc | 62.43±6.91b | 80.41±7.63a |
| Fresh weight of 5 plant roots (g) | 8.41±0.51ab | 7.3±2.01b | 9.82±2.28ab | 7.98±1.91ab | 11.03±2.4a |
| Dry weight of 5 plant roots (g) | 3.41±0.52ab | 3.2±0.77b | 4.11±0.5ab | 3.61±0.85ab | 4.44±0.82a |
| Total fresh weight of peanuts from 5 plants (g) | 159.34±36.12b | 208.81±11.37a | 229.72±28.93a | 215.39±22.11a | 245.35±23.87a |
| Total dry weight of peanuts from 5 plants (g) | 107.7±21.87b | 132.72±17.97ab | 143.08±16.05a | 138.61±12.11a | 145.6±24.17a |
| Seed number of 5 plants | 132.25±21.65c | 162.75±11.44b | 176.5±22.1ab | 171±15.71ab | 187±25.47ab |
| Seeds weight of 5 plants (g) | 79.36±17.61b | 97.81±18.48ab | 105.53±12.45a | 100.99±10.23ab | 101.69±19.28ab |
| Dry weight of 100 peanuts (g) | 143.57±7.04a | 142.65±23.51a | 148.32±10.7a | 114.47±67.31a | 136.93±25.43a |
| 100 Seeds weight (g) | 56.19±3.69a | 57.7±6.71a | 59.97±5.05a | 60.88±3.14a | 55.69±5.75a |
| Kernel yield (%) | 85.92±4.61b | 92.29±1.27ab | 95.05±1.36a | 92.12±1.94ab | 91.3±8.36ab |
| Number of plant branches | 5.5±0.48b | 7.7±0.53a | 7.5±0.48a | 7.6±0.57a | 8.25±0.1a |
| Plant height (cm) | 36.25±2.59c | 36.95±1.68c | 42.43±1.32b | 43.65±3.17b | 48.65±2.25a |
| Average peanut number from one plant | 18.55±4.18b | 21.15±1.68ab | 21.25±2.88ab | 22.05±0.53ab | 23.7±2.44a |
| Average plump peanut number from one plant | 16.65±3.62c | 19±1.13bc | 19.65±2.12abc | 20±1.42abc | 21.9±1.69ab |

Different lowercase letters behind the data represents the significant difference among 5 treatments at *p* < 0.05.
